# Por un abordaje de las desigualdades étnico-raciales y el racismo en el campo de la salud: Un llamado a la equidad

**DOI:** 10.18294/sc.2025.5783

**Published:** 2025-08-06

**Authors:** Andrêa Ferreira, Vivian Carla Honorato dos Santos de Carvalho, Joseph Palumbo

**Affiliations:** 1 Nutricionista. Doctora en Salud Pública. Investigadora senior, Associação de Pesquisa Iyaleta, Bahia, Brasil. andreaferreiracv@gmail.com Associação de Pesquisa Iyaleta Associação de Pesquisa Iyaleta Bahia Brazil andreaferreiracv@gmail.com; 2 Nutricionista. Doctora en Epidemiología. Profesora, Instituto Multidisciplinar em Saúde, Universidade Federal da Bahia, Bahia, Brasil. vivian.honorato@ufba.br Universidade Federal da Bahia Instituto Multidisciplinar em Saúde Universidade Federal da Bahia Bahia Brazil vivian.honorato@ufba.br; 3 Sociólogo. Doctor en Ciencias Sociales. Becario, Consejo de Investigaciones Científicas y Técnicas, con sede en el Centro de Estudios Urbanos y Regionales, Buenos Aires, Argentina. j.palumbo@conicet.gov.ar Consejo Nacional de Investigaciones Científicas y Técnicas Consejo de Investigaciones Científicas y Técnicas Centro de Estudios Urbanos y Regionales Buenos Aires Argentina j.palumbo@conicet.gov.ar

## PRESENTACIÓN

Las intersecciones entre raza y etnia, racismo y salud son un tema urgente en el campo de la salud colectiva. Es ampliamente reconocido el papel fundamental que juega el racismo en estructurar el acceso a derechos básicos de diversas poblaciones y comunidades, moldeando sus contextos de vida y relaciones sociales por medio de procesos históricos y contemporáneos de racialización^1^. El conjunto de artículos aceptados en el marco de la convocatoria “Raza, etnia, racismo y salud”^2^^,^^3^^,^^4^^,^^5^^,^^6^^,^^7^^,^^8^ proponen examinar las maneras en que cuestiones vinculadas a la raza/etnia y el racismo condicionan procesos de salud-enfermedad-atención-cuidado para individuos y grupos racializados, proporcionando nuevas evidencias empíricas y contribuyendo a perspectivas emergentes. 

El racismo es un fenómeno que se expresa en múltiples niveles: aunque puede manifestarse a nivel interpersonal, como actos cotidianos discriminatorios o prejuicios arraigados, también existe a nivel estructural, conocido como el “racismo institucional”, en tanto se configuran normas, sistemas e instituciones de manera tal que favorecen a los grupos dominantes, reforzando y reproduciendo así las desigualdades raciales y étnicas^9^. El racismo tiene impactos profundamente dañinos en la vida cotidiana de grupos que son vulnerados por ese sistema, determinando sus posibilidades de nacer, crecer, vivir y morir. Cabe destacar que el racismo es un determinante social estructural de la salud que produce jerarquizaciones sociales en las cuales un grupo será permanentemente privilegiado -en este caso, la población blanca- en detrimento de la población negra e indígena, que sufrirán los impactos de tal vulneración en todos los ámbitos de sus vidas^9^. Asimismo, en muchos contextos, los impactos del racismo institucional han sido invisibilizados por la negación generalizada de su existencia, promoviendo la naturalización de las desigualdades resultantes, a menudo vistas como problemas culturales o económicos, sin reconocer el papel fundamental del racismo en las desigualdades sociales que profundizan la vulnerabilidad de esas poblaciones^10^. Raza es una construcción social falsamente diseminada e históricamente estructurada en torno a marcadores fenotípicos (como color de piel), con la finalidad de crear y mantener jerarquías sociales y relaciones de poder y dominación^9^. Por lo tanto, más que poner el foco en la noción de raza en sí, se debe atender a la *racialización* como proceso, a través del cual los grupos sociales dominantes construyen las categorías raciales, que luego serán impuestas a los grupos oprimidos. Si bien son categorías que a veces son tomadas como intercambiables, raza y etnia son conceptualmente distinguibles, siendo la etnia un constructo multidimensional que se asocia con aspectos como las tradiciones, las costumbres y la cultura. 

## RACIALIZACIÓN

El concepto de racialización es útil para echar luz sobre los procesos según los cuales estas desigualdades se estructuran en distintos contextos históricos y geográficos. Dicho proceso se ha desarrollado según trayectorias históricas específicas en diversas regiones del mundo -tanto del “norte” como en el “sur” global- y, por lo tanto, es necesario examinar las particularidades de los procesos sociales, culturales, económicos y políticos que estructuran las relaciones de poder y las formas de dominación racial/étnica en estos distintos contextos, así como las desigualdades persistentes causadas por el racismo y sus manifestaciones. Por ejemplo, aunque América Latina es una región reconocida por su diversidad cultural, el legado del colonialismo estableció definiciones de humanidades y creó jerarquías raciales y étnicas en las Américas. Poblaciones indígenas y afrodescendientes -que representan una proporción significativa de la población de la región- se encuentran desproporcionadamente afectados por la pobreza y el acceso limitado a la educación y la salud^11^. En otras regiones marcadas por historias de colonialismo, las relaciones raciales y étnicas han sido moldeadas, entre otros factores, por el desplazamiento forzado, el genocidio y la invisibilización de los pueblos originarios en América del Norte y Australia, así como por el legado de la esclavitud y la segregación en EEUU. Las jerarquías raciales y los sistemas de explotación institucionalizados a través del colonialismo europeo en el continente africano continúan teniendo implicancias devastadoras hasta el día de hoy. Las secuelas del apartheid en Sudáfrica, por ejemplo, han sostenido profundas disparidades raciales que afectan negativamente a las personas negras sudafricanas^12^. En el sur de Asia, la herencia del sistema de castas ha funcionado como un proceso de racialización, dando lugar a marcadas desigualdades y formas de discriminación hacia grupos racializados^13^. En la Europa contemporánea, la racialización de grupos como los pueblos romaníes y las personas migrantes provenientes de antiguas colonias se encuentra moldeada por dinámicas cruzadas de xenofobia, islamofobia y exclusión cultural, entre otras^14^.

## INEQUIDADES RACIALES/ÉTNICAS

En diversas regiones del mundo, los impactos de estos procesos son evidentes en las desigualdades en salud que afectan a las poblaciones racializadas de manera desproporcionada^15^. La intersección entre raza, etnia y salud revela, por lo tanto, una realidad preocupante: el racismo estructural y las inequidades raciales/étnicas continúan marginando y negando el acceso a derechos básicos y fundamentales a las comunidades negras e indígenas, lo que agrava las desigualdades en salud y perpetúa ciclos de negación de derechos, pobreza y exclusión. Estos contrastes no son únicamente el resultado de circunstancias individuales, sino que están profundamente arraigados en las estructuras de la sociedad -incluso, en los sistemas de salud- que, a menudo, constituyen barreras en el acceso a los servicios de salud y en la calidad de la atención brindada a personas racializadas, vinculadas a sesgos raciales implícitos y una falta de reconocimiento de las necesidades específicas de estas comunidades. 

Además de los contextos de vida de las personas negras en el sistema mundo en general, resulta relevante discutir su distribución geográfica en las grandes ciudades del sur global, que son fuertemente marcadas por la segregación residencial racial y por vacíos asistenciales, que relega a las personas negras a los márgenes de las ciudades y en territorios desprovistos de servicios sanitarios^10^. La segregación genera condiciones adversas para la salud, ya que los lugares donde reside la mayoría de las personas negras han sido históricamente precarizados, con viviendas inadecuadas en relación con las condiciones estructurales, sin acceso a servicios básicos de saneamiento, agua potable y equipamientos de salud, como puestos sanitarios, farmacias, parques y espacios de recreación. Todo esto contribuye a una mayor exposición a factores de riesgo, además de los elevados índices de violencia, lo que resulta en una acumulación de desventajas en términos de salud^9^. Williams y Collins^16^ consideran la segregación residencial racial una causa fundamental de las disparidades raciales en salud, dado que es una de las principales causas de la desigualdad en relación con el estatus socioeconómico, determinando el acceso a la educación, el empleo y las oportunidades. La segregación de los barrios en los que residen las personas negras les impide el acceso a recursos y bienes, además de facilitar su control político y explotación económica^10^^,^^17^.

Las desigualdades raciales y étnicas en salud también se hacen evidentes en el acceso diferencial que tienen las poblaciones y comunidades negras e indígenas a infraestructuras y servicios sanitarios. La accesibilidad geográfica a los servicios de salud es apenas un aspecto de esto. Es ampliamente reconocido que el concepto de “acceso” no solo remite a la dimensión espacial de la existencia o disponibilidad de los servicios sanitarios, sino que incluye otros factores sociales que pueden favorecer u obturar su utilización efectiva^18^^,^^19^. Por ejemplo, en países con sistemas de salud fragmentados y/o privatizados, las poblaciones racializadas tienden a poseer menores niveles de cobertura de salud o acceso limitado a los servicios sanitarios de mayor calidad^20^^,^^21^. La asociación entre segregación residencial racial y los riesgos ambientales y sociales también ha sido examinada con relación a resultados de salud desfavorables^22^^,^^23^.

Los datos epidemiológicos desagregados por raza/etnia evidencian estos contrastantes resultados en salud en numerosos contextos nacionales o regionales. Un gran número de estudios provenientes de EEUU ha explorado la dimensión racial/étnica de la atención y los resultados en salud^24^^,^^25^, mostrando brechas raciales en todos los grupos etarios en relación con enfermedades cardiovasculares, VIH-sida, mortalidad infantil y materna, entre otros^26^^,^^27^. La pandemia de covid-19 también dejó al descubierto las implicancias perdurables de las desigualdades raciales arraigadas en EEUU^10^^,^^28^. 

Brechas similares en términos de salud existen en muchas regiones del mundo, y aunque las problemáticas específicas pueden variar, los datos tienden a revelar desventajas consistentes para comunidades racializadas^29^. En América Latina -por ejemplo, en países como Guatemala y Bolivia- las tasas de mortalidad materna entre mujeres indígenas son más altas que los promedios nacionales, mientras que comunidades afrodescendientes en Brasil y Colombia experimentan tasas más altas de enfermedades crónicas como hipertensión y diabetes, a menudo vinculadas al acceso inadecuado a cuidados preventivos y condiciones de vida saludables^10^^,^^30^^,^^31^.

El racismo en la atención en salud no siempre se manifiesta de forma abierta; a menudo, se expresa de manera más sutil y sistémica. Por ejemplo, la falta de una atención culturalmente competente -es decir, servicios de salud que respeten e integren las creencias, prácticas y lenguas de diversas poblaciones- genera barreras para el acceso a tratamientos efectivos. Las comunidades afrodescendientes pueden ser objeto de estereotipos que derivan en diagnósticos erróneos o tratamientos inadecuados. Estas fallas estructurales no solo afectan a las personas de manera directa, sino que también deterioran la confianza en los sistemas de salud, lo cual puede desalentar a los grupos marginados a buscar atención médica^32^. Asimismo, la intersección entre raza/etnia y otras formas de desigualdad social -como el género, el estatus socioeconómico, la edad, entre otras- agravan estas disparidades^33^. 

Frente a estas desigualdades, existe la imperante necesidad de seguir generando conocimiento sobre las disparidades raciales y étnicas en materia de salud. Para tal fin, resulta necesario no solo elaborar análisis que tomen en cuenta la dimensión racial de las desigualdades en salud, sino también la producción de datos primarios que permitan llevar adelante dichos análisis^34^. El conjunto de artículos resultantes de la convocatoria “Raza, etnia, racismo y salud” realiza un aporte en ese sentido, a partir de evidencias empíricas desde América Latina y Europa, con estudios de Argentina, Brasil, Chile, México y España.

En primer lugar, en línea con la literatura existente, varias contribuciones analizan el racismo tanto individual como institucional como una barrera significativa en procesos de salud-enfermedad-atención-cuidado, exacerbando la vulnerabilidad de poblaciones racializadas. Por ejemplo, los efectos psicológicos perjudiciales de lidiar con el racismo pueden ser notados en un estudio sobre las trayectorias de atención en salud mental de jóvenes indígenas en Oaxaca, México^5^. Aun cuando la migración interna rural-urbano facilitó el acceso a servicios de salud mental, la interpretación de sus experiencias mediante una lente “occidental” se convirtió en una barrera a la atención efectiva y, en algunos casos, redundó en una sensación de alienación cultural, impactando en la salud mental de estos jóvenes. Otro de los estudios examina las tendencias en el uso de psicofármacos entre comunidades indígenas y *quilombolas* en Brasil, y muestra que el uso de antidepresivos y ansiolíticos fue más prevalente entre mujeres socialmente vulnerables, llevando a sus autores a sugerir que “la fragilidad económica y las precarias condiciones de vida de estos territorios […] probablemente actúan como importantes factores de determinación social del sufrimiento psíquico y del uso de psicofármacos”^7^. De manera similar, los procesos de racialización vinculados a flujos migratorios internacionales pueden incidir en la salud mental y bienestar de personas migrantes en muchas formas. Además de enfrentar xenofobia y prejuicios con relación a su estatus migratorio, grupos racializados deben lidiar con lo que una de las autoras denomina “trauma racial” -o los impactos de convivir con las disparidades causadas por el racismo estructural- que ilustra al analizar las consecuencias en salud mental de las experiencias relacionadas de migración y racismo entre la población de origen afrocaribeña en España^3^. Las implicancias para la salud de la dimensión racial de los flujos migratorios también se hacen evidentes en un estudio sobre trabajadores y trabajadoras migrantes en el sector hortícola de La Plata, Argentina, en el que la autora muestra cómo raza/etnia opera como un factor estructurante en la inserción laboral de estas personas migrantes, quienes son sometidas a formas intensivas de explotación laboral y expuestas a factores de riesgo ocupacional^8^. 

Otro tema preponderante tiene que ver con la pertenencia cultural de la atención en salud, analizada en contribuciones de esta convocatoria. Además de aportar evidencia de la necesidad de una atención sanitaria culturalmente pertinente y adecuada, los artículos exploran la complejidad de llevar esto a la práctica. Por ejemplo, un análisis de la fisioterapia con personas del pueblo mapuche en Chile revela las dificultades de alcanzar una atención culturalmente pertinente, aun frente a la existencia de un programa de salud indígena diseñado desde una perspectiva intercultural, en función de factores como la falta de diálogo real entre el personal médico y el programa de salud indígena y sesgos epistémicos que priorizan los supuestos de la biomedicina occidental, a la vez que marginalizan las perspectivas indígenas^4^. Problemáticas similares se evidenciaron con relación al análisis previamente comentado de los servicios de salud mental para jóvenes indígenas en México, cuando profesionales de la salud mental no lograron contemplar el papel de la discriminación racial/étnica ni la necesidad de adoptar una perspectiva intercultural; incluso, en algunos casos, los mismos individuos relativizaron la relevancia de estos factores en su salud mental^5^.

Por último, e intrínsecamente vinculado a lo anterior, varios artículos de esta convocatoria dejan al descubierto la necesidad de seguir produciendo evidencias sobre experiencias con diferentes formas de práctica antirracista en los procesos de salud-enfermedad-atención-cuidado. Por ejemplo, un estudio proveniente de Brasil sobre un programa de educación permanente destinado a profesionales del Sistema Único de Salud muestra cómo tales iniciativas constituyen una “estrategia poderosa para fomentar el pensamiento crítico sobre el tema, así como para delinear posibles estrategias para combatir el racismo dentro de los servicios de salud”, a pesar de los desafíos y retrocesos que pueden surgir en la implementación^6^. Con frecuencia, la práctica antirracista en el ámbito de la salud surge a partir de las demandas y la organización de las propias comunidades racializadas. 

Esto se vislumbra en un estudio sobre las respuestas a la pandemia de covid-19 entre comunidades diaguitas en la provincia argentina de Catamarca, donde la acción comunitaria e intercomunitaria por parte de organizaciones indígenas fue clave para atenuar los efectos de la pandemia^2^. 

Como un todo, los artículos resultantes de la convocatoria “Raza, etnia, racismo y salud” iluminan la necesidad de contribuir con diversas perspectivas globales al conocimiento sobre las intersecciones entre raza/etnia y salud, incluyendo las múltiples manifestaciones del racismo institucional en la atención sanitaria que experimentan día a día las poblaciones negras e indígenas alrededor del mundo. Así, estos trabajos no solo realizan un aporte a debates académicos sobre estas problemáticas, sino también se convierte en un llamado para la equidad.

## References

[1] Paradies Y, Ben J, Denson N, Elias A, Priest N, Pieterse A (2015). Racism as a determinant of health: A systematic review and meta-analysis. PLOS ONE.

[2] Guerreiro LG (2024). Covid-19 y comunidades diaguitas: desigualdades estructurales y estrategias (inter)comunitarias en el departamento Santa María (Catamarca, Argentina). Salud Colectiva.

[3] Tineo Durán JC (2024). Geografías del (des)amparo: Bosquejos en salud mental antirracista. Salud Colectiva.

[4] Manríquez-Hizaut M, Martínez-Campos T, Lagos-Gallardo N, Rebolledo-Sanhueza J (2024). Desafíos para la pertinencia cultural de fisioterapia en la atención de población mapuche en Chile. Salud Colectiva.

[5] Bandala MAS, Sesia PM (2024). Racism and mental health among indigenous youth living in the Metropolitan Area of Oaxaca, Mexico. Salud Colectiva.

[6] Ferreira MGM, Jesus GM de, Silva EKP da, Silva VB, Silva MC da (2024). Educación permanente en salud para directivos como herramienta para combatir el racismo institucional en un municipio del noreste de Brasil. Salud Colectiva.

[7] Loures GSG, Ronzani TM, Paula PAB, Dimenstein M, Fernandes SL, Leite JF (2024). Uso de psicofármacos entre comunidades indígenas y quilombolas en Brasil. Salud Colectiva.

[8] Caimmi N (2024). La desigualdad racial de la producción de hortalizas en fresco y flores de corte en Argentina: análisis en el Gran La Plata. Salud Colectiva.

[9] Werneck J (2016). Racismo institucional e saúde da população negra. Saúde e Sociedade.

[10] Goes EF, Ramos DDO, Ferreira AJF (2020). Desigualdades raciais em saúde e a pandemia da Covid-19. Trabalho, Educação e Saúde.

[11] Perreira KM, Telles EE (2014). The color of health: Skin color, ethnoracial classification, and discrimination in the health of Latin Americans. Social Science & Medicine.

[12] Charasse-Pouélé C, Fournier M (2006). Health disparities between racial groups in South Africa: A decomposition analysis. Social Science & Medicine.

[13] Thapa R, van Teijlingen E, Regmi PR, Heaslip V (2021). Caste exclusion and health discrimination in South Asia: A systematic review. Asia-Pacific Journal of Public Health.

[14] Pattillo M, Stieglitz S, Angoumis K, Gottlieb N (2023). Racism against racialized migrants in healthcare in Europe: a scoping review. International Journal for Equity in Health.

[15] White K, Lawrence JA, Tchangalova N, Huang SJ, Cummings JL (2020). Socially-assigned race and health: a scoping review with global implications for population health equity. International Journal for Equity in Health.

[16] Williams DR, Collins C (2020). Racial residential segregation: a fundamental cause of racial disparities in health. Public Health Reports.

[17] Kilomba G (2019). Memórias da plantação: episódios de racismo cotidiano.

[18] Khan AA, Bhardwaj SM (1994). Access to health care: A conceptual framework and its relevance to health care planning. Evaluation & the Health Professions.

[19] Aveni SM (2019). El acceso a la salud: una revisión conceptual interdisciplinaria. Sudamérica: Revista de Ciencias Sociales.

[20] Viáfara-López CA, Palacios-Quejada G, Banguera-Obregón A (2021). Inequidad por la condición étnico-racial en el aseguramiento de salud en Colombia: un estudio de corte transversal. Revista Panamericana de Salud Pública.

[21] Lee DC, Liang H, Shi L (2021). The convergence of racial and income disparities in health insurance coverage in the United States. International Journal for Equity in Health.

[22] Richardson LD, Norris M (2010). Access to health and health care: How race and ethnicity matter. Mount Sinai Journal of Medicine.

[23] Barber S, Diez Roux AV, Cardoso L, Santos S, Toste V, James S (2018). At the intersection of place, race, and health in Brazil: Residential segregation and cardio-metabolic risk factors in the Brazilian Longitudinal Study of Adult Health (ELSA-Brasil). Social Science & Medicine.

[24] LaVeist TA, Isaac LA (2013). Race, Ethnicity, and Health: A Public Health Reader.

[25] Feagin J, Bennefield Z (2014). Systemic racism and U.S. health care. Social Science & Medicine.

[26] Macias-Konstantopoulos WL, Collins KA, Diaz R, Duber HC, Edwards CD, Hsu AP (2023). Race, healthcare, and health disparities: A critical review and recommendations for advancing health equity. Western Journal of Emergency Medicine.

[27] Javed Z, Haisum Maqsood M, Yahya T, Amin Z, Acquah I, Valero-Elizondo J (2022). Race, racism, and cardiovascular health: Applying a social determinants of health framework to racial/ethnic disparities in cardiovascular disease. Circulation: Cardiovascular Quality and Outcomes.

[28] Canales AI, Castillo Fernández D (2022). Desigualdad social y étnico-racial frente a la covid-19 en Estados Unidos. Migración y Desarrollo.

[29] Cormack DM, Harris RB, Stanley J (2013). Investigating the relationship between socially-assigned ethnicity, racial discrimination and health advantage in New Zealand. PLOS ONE.

[30] Mena-Meléndez L (2020). Ethnoracial child health inequalities in Latin America: Multilevel evidence from Bolivia, Colombia, Guatemala, and Peru. SSM - Population Health.

[31] Paulino NA, Vázquez MS, Bolúmar F (2019). Indigenous language and inequitable maternal health care, Guatemala, Mexico, Peru and the Plurinational State of Bolivia. Bulletin of the World Health Organization.

[32] Ben J, Cormack D, Harris R, Paradies Y (2017). Racism and health service utilisation: A systematic review and meta-analysis. PLOS ONE.

[34] Schulz AJ, Mullings L (2006). Gender, race, class, and health: Intersectional approaches.

[35] Carabali M, Barber S, Ferreira AJF, Ortigoza AF, Ramos D, Goes E (2024). Pan-American data initiative for the analysis of population racial/ethnic health inequities: the Pan-DIASPORA project. The Lancet Regional Health - Americas.

