# Supplementary material for: Por un abordaje de las desigualdades étnico-raciales y el racismo en el campo de la salud: Un llamado a la equidad
Source: Salud Colect. 2025 Aug 6;21:e5783. [Article in Spanish] doi: 10.18294/sc.2025.5783 (PMC12718098; doi:10.18294/sc.2025.5783)
Supplement: Translation

# Translation: Addressing racial/ethnic disparities and racism in health: A call for equity

**Authors:** Andrêa Ferreira, Vivian Carla Honorato dos Santos de Carvalho, Joseph Palumbo

**Affiliations:** 1 Nutritionist. Doctor of Public Health. Senior researcher, Associação de Pesquisa Iyaleta, Bahia, Brasil. andreaferreiracv@gmail.com; 2 Nutritionist. Doctor in Epidemiology. Professor, Instituto Multidisciplinar em Saúde, Universidade Federal da Bahia, Bahia, Brasil. vivian.honorato@ufba.br; 3 Sociologist. Doctor in Social Sciences. Fellow, Consejo de Investigaciones Científicas y Técnicas, based in Centro de Estudios Urbanos y Regionales, Buenos Aires, Argentina. j.palumbo@conicet.gov.ar

## PRESENTATION

The intersections between race and ethnicity, racism, and health are a matter of pressing concern in the field of collective health. It is widely recognized that racism plays a fundamental role in structuring access to basic rights for diverse communities and populations, shaping their living contexts and social relations through historical and contemporary racialization processes^1^. The articles accepted as part of the call for papers on “Race, ethnicity, racism, and health”^2^^,^^3^^,^^4^^,^^5^^,^^6^^,^^7^^,^^8^^)^ intend to examine the ways in which issues related to race/ethnicity and racism condition health-disease-care-prevention processes for racialized individuals and groups, offering new empirical evidence and contributing to emerging perspectives. 

Racism is a phenomenon that is evident at multiple scales: although it can manifest at the interpersonal level as day-to-day acts of discrimination or ingrained prejudiced attitudes, it also exists at the structural level - also known as “institutional racism” - in norms, systems, and institutions that are configured in such a way as to favor dominant groups, thereby reinforcing and reproducing racial/ethnic inequalities^9^. Racism has profound and harmful impacts in the everyday lives of individuals belonging to groups that are made vulnerable in this system, shaping their conditions of birth, growth, life, and death. Therefore, it should be emphasized that racism is a structural social determinant of health, producing social hierarchies in which one group - white populations, in this case - is permanently privileged at the expense of Black, Brown, and Indigenous populations, who experience vulnerability in all aspects of life^9^. Furthermore, in many contexts, the impacts of institutional racism have been made invisible through the widespread denial of its existence, which promotes the naturalization of resulting inequalities. These inequalities are often interpreted as cultural or economic problems, without acknowledging the fundamental role racism plays in the social disparities that deepen the vulnerability of these populations^10^. Race is a social construct, falsely disseminated and historically structured around phenotypic markers (such as skin color), with the purpose of creating and sustaining social hierarchies and power relations^9^. Therefore, rather than limiting focus to race in and of itself, attention should be paid to *racialization* as a process whereby racial categories are constructed by dominant social groups and imposed on oppressed groups. Although they are often seen as interchangeable, race and ethnicity are conceptually distinguishable, as ethnicity refers to a multidimensional construct associated with traditions, customs, and culture. 

## RACIALIZATION

The concept of racialization is useful for shining light on the process by which these inequalities are structured in different historical and geographical contexts. This process has unfolded according to specific historical trajectories in diverse regions throughout the world - both in the Global “North” and “South” - and it is therefore necessary to examine the specificities of social, cultural, economic, and political processes that structure power relations and forms of racial/ethnic domination in different contexts, as well as the persistent inequalities caused by racism and its manifestations. For example, although Latin America is a region known for its cultural diversity and rich heritage, the legacy of colonialism established the definitions of the Humanities and created racial and ethnic hierarches in American societies. Indigenous and Afro-descendant populations - who make up a significant portion of the region’s population - are disproportionately affected by poverty and limited access to education and healthcare^11^. In other regions marked by histories of colonialism, racial and ethnic relations have been shaped - among other factors - by the displacement, genocide, and erasure of Indigenous peoples in North America and Australia, or the legacy of slavery and segregation in the United States. Racial hierarchies and exploitative systems institutionalized through European colonialism on the African continent continue to have devastating implications to this day. The aftermath of apartheid in South Africa, for example, has upheld ingrained racial disparities that negatively impact Black South Africans^12^. In South Asia, the legacy of the caste system has functioned as a racialization process, resulting in marked disparities and discrimination faced by racialized groups^13^. In contemporary Europe, the racialization of groups such as the Romani people and migrants from former colonies is shaped by intersecting dynamics of xenophobia, Islamophobia, and cultural exclusion, among others^14^. 

## RACIAL/ETHNIC INEQUITIES

Across world regions, the impacts of these processes are visible in health disparities that disproportionately affect populations impacted by racism and its multiple manifestations^15^. Therefore, the intersection of race, ethnicity, and health reveals a troubling reality: structural racism and racial/ethnic inequities continue to marginalize and deny access to basic and fundamental rights to Black and Indigenous communities, exacerbating health inequities and perpetuating cycles of rights denial, poverty, and exclusion. These disparities are not merely the result of individual circumstances, but are deeply rooted in the structures of society - including within healthcare systems - which often create barriers to both access to services and the quality of care provided to racialized individuals. These barriers are frequently linked to implicit racial bias and to a lack of recognition of the specific needs of these communities.

Beyond the general situation of Black and Brown peoples in the world system, it is necessary to examine their spatial distribution in major cities of the Global South, characterized by racial residential segregation and significant gaps in healthcare provision, where these groups are pushed to the urban peripheries that often lack adequate health services^10^. Segregation also generates adverse health conditions, as the areas where the majority of Black populations reside have historically been subject to neglect. These neighborhoods typically suffer from inadequate housing conditions and lack access to basic sanitation, clean water, and essential healthcare infrastructure such as clinics, pharmacies, parks, and recreational spaces. This contributes to greater exposure to risk factors and high levels of violence, ultimately resulting in a cumulative burden of health issues^9^. Williams and Collins^16^ identify racial residential segregation as a fundamental cause of racial health disparities, as it is a primary driver of inequalities in socioeconomic status, shaping access to education, employment, and opportunities. Neighborhood segregation limits the access of Black and Brown communities to resources and assets, while also facilitating the political control and economic exploitation of these communities^10^^,^^17^.

Racial and ethnic health inequalities are also visible in the differential access that Black, Brown, and Indigenous populations have to healthcare services and infrastructure. Geographical accessibility to healthcare services is just one aspect of this. It is widely recognized that the concept of “access” is not limited to the spatial dimension of the existence or availability of healthcare services, but also includes social factors that can facilitate or hinder their effective utilization^18^^,^^19^. For example, in countries with fragmented or privatized healthcare systems, racialized populations often have lower levels of healthcare coverage or limited access to higher-quality healthcare services^20^^,^^21^. Racial residential segregation has also been associated with exposure to social and environmental risk factors, linked to adverse health outcomes^22^^,^^23^.

Epidemiological data disaggregated by race/ethnicity provide evidence of these disparities in health outcomes across national and regional contexts. A great deal of research from the United States has explored the significance of race/ethnicity in healthcare and health outcomes^24^^,^^25^, showing racial/ethnic gaps across all age groups with regards to various outcomes such as cardiovascular disease, HIV/AIDS, infant and maternal mortality, among others^26^^,^^27^. The covid-19 pandemic was also telling in terms of the ongoing implications of these deep-seated racial inequalities in contexts such as Brazil and the United States^10^^,^^28^. 

Similar health gaps exist in many world regions; although the specific issues may vary, data tend to show ingrained disadvantages for Black, Brown, and Indigenous communities^29^. In Latin America, for example in countries like Guatemala and Bolivia, maternal mortality rates among Indigenous women are significantly higher than national averages, while Afro-descendant communities in Brazil and Colombia face higher rates of chronic diseases such as hypertension and diabetes, often linked to inadequate access to preventive care and healthy living conditions^10^^,^^30^^,^^31^. 

Racism in healthcare is not always overt; it often manifests in subtler, more systemic ways. For instance, the lack of culturally competent care - health services that respect and integrate the cultural beliefs, practices, and languages of diverse populations - creates barriers to effective treatment. Afro-descendant communities may encounter stereotypes that lead to misdiagnosis or inadequate treatment. These systemic failures not only harm individuals but also erode trust in healthcare systems, discouraging marginalized groups from seeking care altogether^32^. Furthermore, the intersection of race and ethnicity with other forms of social inequality - gender, socioeconomic status, age, among others - compound these disparate outcomes^33^. 

In light of these inequalities, there is a critical need for the continued production of knowledge on racial and ethnic disparities in health. To that end, it is not only necessary to carry out analyses that take into account the racial dimension of health inequalities, but also the production of primary data that would allow for such analyses^34^. The group of articles stemming from the call for papers on “Race, ethnicity, racism, and health” make a contribution in this regard, providing empirical evidence from Latin America and Europe, with studies from Argentina, Brazil, Chile, Mexico, and Spain.

First of all, in line with the existing literature, several of the articles analyze both individual-level and institutional racism as a significant barrier in health-disease-care-prevention processes, exacerbating the vulnerability of racialized populations. For example, the detrimental psychological impacts of dealing with racism are evident in a study of mental health care trajectories of Indigenous youth in Oaxaca, Mexico^5^. Even when internal rural-urban migration facilitated access to mental health services, the filtering of their experiences through a “Westernized” lens became a barrier to effective care and in some cases led to a sense of cultural alienation, impacting the mental health of these young people. Another of the studies examines trends in psychopharmaceutical use among Indigenous and *quilombola* communities in Brazil, showing that the use of antidepressants and anxiolytics was most prevalent among socially vulnerable women, thereby suggesting that “the economic vulnerability and precarious living conditions in these territories [...] likely function as significant social determinants of psychological distress and the use of psychopharmaceuticals”^7^. Similarly, racialization processes surrounding international migratory flows can also influence mental health and wellbeing in multiple ways. In addition to facing xenophobia and prejudice with regards to their migratory status, racialized groups must cope with what one of the authors calls “racial trauma” - or the psychological impacts of living with the disparities caused by structural racism - as illustrated through the mental health consequences of the dual experiences of migration and racism in a population of Afro-Caribbean origin in Spain^3^. The health implications of the racial dimension of migration flows is also apparent in the study on migrant workers in the agricultural sector in La Plata, Argentina, where the author evidences how race/ethnicity functions as a structuring factor in the labor market integration of these migrants, who are subjected to intensive labor exploitation and exposed to occupational risk factors^8^. 

Another prominent theme has to do with the cultural competence of healthcare, analyzed in several articles accepted as part of this call for papers. Beyond just contributing further evidence of the need for culturally sensitive and appropriate care, several articles included in the collection explore the complexities of putting this into practice. For example, an analysis of physiotherapy rehabilitation with Mapuche individuals in Chile reveals the difficulties in achieving culturally sensitive care even in the presence of an Indigenous health program devised from an intercultural perspective, resulting from factors such as a lack of meaningful dialogue between healthcare providers and the Indigenous health program or epistemic biases favoring Western biomedical assumptions and marginalizing Indigenous perspectives^4^. Similar issues also appeared in relation to the previously mentioned analysis of mental health services for Indigenous youth in Mexico, where mental health care providers were often unable to account for both the role of racial/ethnic discrimination and the need for an intercultural perspective, in some cases leading the individuals themselves to downplay the relevance of these factors in conditioning their mental health^5^. 

Lastly, and closely related to the previous issue, several articles make a case for the need to produce further evidence on experiences with different forms of antiracist practice in health-disease-care-prevention processes. For example, a study conducted in Brazil on a continuing education program for professionals in the Unified Health System demonstrates how such initiatives represent “powerful strategies for encouraging critical thinking on the issue and outlining possible strategies to combat racism in healthcare services,” despite the challenges and setbacks that may arise during their implementation^6^. More often than not, antiracist practice in healthcare stems from the demands and organization of racialized communities themselves. This is evident in an article examining the responses to the Covid-19 pandemic among Diaguita communities in the province of Catamarca, in northern Argentina, where community and inter-community action led by Indigenous organizations played a crucial role in mitigating the effects of the pandemic^2^. 

Taken together, the articles published as a result of the call for papers on “Race, ethnicity, racism, and health” point to the need to contribute diverse, global perspectives to the body of knowledge on the intersections between race/ethnicity and health, including the multilevel manifestations of institutional racism in healthcare that Black, Brown, and Indigenous populations around the world experience on a daily basis. Therefore, these articles not only function as a contribution to scholarly debates on these issues, but also as a call for equity.
